# Understanding Brains: Details, Intuition, and Big Data

**DOI:** 10.1371/journal.pbio.1002147

**Published:** 2015-05-12

**Authors:** Eve Marder

**Affiliations:** Volen Center and Biology Department, Brandeis University, Waltham, Massachusetts, United States of America

## Abstract

Understanding how the brain works requires a delicate balance between the appreciation of the importance of a multitude of biological details and the ability to see beyond those details to general principles. As technological innovations vastly increase the amount of data we collect, the importance of intuition into how to analyze and treat these data may, paradoxically, become more important.

## Abstract 2

This personal view from a pioneer of connectomics asks how we should balance the plethora of biological data with the need to extract general principles that bring insight into brain function.

This Essay is part of the "Where Next?" Series.

Experimental biologists collect details. In the early days, naturalists prowled their backyards, local forests, and meadows. They traveled the Amazon River and African savannahs and collected species and categorized them. These collectors of beetles and ferns then tried to formulate hypotheses about evolutionary relationships by looking at commonalities of structure, function, and development. In those days, there was an implicit belief that the passionate acquisition of detailed information about the idiosyncrasies of individual species contained the route to understanding the general principles of life. Although today’s experimental neuroscientists employ much more sophisticated methods, most retain a deep conviction that the specific properties of molecules, synapses, neurons, circuits, and connectomes are important for understanding how brains, be they small or large, work.

Modern neuroscience traces much of its history to prescient physiologists, pharmacologists, and anatomists. Early anatomists such as Ramón y Cajal pioneered the use of stains to reveal the structure of neurons and to make astonishing leaps of intuition about the structure and function of brain circuits [1]. Early physiologists and pharmacologists deduced the existence of receptors and kinetics from bioassays [2,3]. Observation and reasoning from first principles led T. Graham Brown [4,5] to first articulate that reciprocal inhibition in the spinal cord could underlie the generation of rhythmic movements. Cajal and Brown anticipated systems neuroscience as we know it today: understanding how the particular properties of neurons and their connections give rise to the complex and adaptive responses that allow animals to interact with each other and their worlds.

## Connectomics

The study of how the dynamics of small circuits depend on the intrinsic properties of neurons and their synaptic connectivity [6–9] started almost 50 years ago when intracellular recording and stimulating techniques, coupled with intracellular dye fills [10,11], produced some of the first wiring diagrams, or what we now call connectomes. During this early era of “circuit cracking,” investigators worked hard to identify neurons and determine their connectivity, because it was clear, even then, that a connectivity diagram was absolutely necessary to understand circuit dynamics.

By the early 1980s, Judith Eisen and I realized the presence of electrical synapses and other polysynaptic connections could create parallel pathways, or multiple routes for information travel throughout a circuit. We were studying the interactions among the neurons of the pyloric circuit in the stomatogastric ganglion of the lobster *Panulirus interruptus* when we realized to our horror that there were nine possible circuits connecting the AB (anterior burster), PD (pyloric dilator), and LP (lateral pyloric) neurons that were all consistent with the intracellular recordings we had made [12]. In this case, because of the small numbers of neurons involved, we were able to disambiguate the circuit by photoinactivating individual neurons or pairs of neurons in order to determine the actual circuit [12]. This revealed that the LP neuron evoked inhibitory postsynaptic potentials (IPSPs) seen in the AB neuron are not direct but are instead a consequence of their electrical coupling with the PD neurons. Does the actual circuit matter, or would any of the nine possible circuits provide sufficient insight into how the circuit works? To a first approximation, one might think these are all functionally equivalent. However, on closer examination, knowing the actual circuit becomes important for understanding how the LP regulates the bursting dynamics of the AB and PD neurons.

Knowing the “true connectome” does not free one from the confounds produced by parallel pathways in circuits. Gutierrez et al. [13] constructed a simple circuit model composed of only five neurons, loosely motivated by the connectivity of the crab stomatogastric ganglion. As the strengths of the chemical and electrical synapses in the circuit are modified, the circuit can produce a wide variety of behaviors [13]. This by itself tells us that knowing the anatomical connectivity merely gives us the necessary starting conditions for understanding how the circuit works. In this example, because of the parallel pathways, again produced by electrical coupling, there are three entirely different circuit mechanisms that can produce almost identical changes in circuit dynamics [13]. Thus, it is impossible to infer circuit structure from a manipulation that produces a change in circuit output. Without knowing the connectome, one would be hard-pressed to predict the existence of these parallel pathways. That said, the connectome itself is once again only a starting point for understanding circuit dynamics. As valuable as the new connectome initiatives [14–16] will be for understanding medium and large circuits, they will only allow us to formulate hypotheses about how these circuits might work. Careful and thoughtful work will be necessary to reveal the multitude of ways in which each specific circuit operates.

## Facing the Waves of Big Data

Each technological advance in neuroscience has brought a wealth of new knowledge. Today, neuroscience is benefitting from an unprecedented explosion of new technologies, including genomics, optogenetics, mass spectroscopy, and multielectrode recording, that have thrust us into a qualitatively different regime of data analysis from any that we have hitherto experienced.

Generations of systems electrophysiologists patiently studied brains with single units, recorded one electrode at a time, and deduced some of the fundamental principles of receptive fields [17]. Today, people routinely use multi-electrode arrays, and some dream of simultaneously recording from hundreds of thousands or more electrodes in awake animals doing interesting tasks [18]. Likewise, for years, molecular and developmental neurobiologists diligently searched for molecules one at a time that are important for synapse function [19] and development [20,21]. Today, we are collecting data on the expression of thousands of genes in single brain regions or identified neurons [22,23].

Thus, we are not anticipating the era of “big data.” We are already surfing or wiping out in waves of data arriving with hurricane force. Our more quantitative colleagues are developing new tools designed to extract meaning from the giant sets of possible correlations and connections in the very large data sets we now collect. But to someone like myself who has found understanding the dynamics and modulation of small circuits challenging [7,8], I view the future with a mix of tremendous excitement and serious trepidation.

The reasons for excitement are obvious: new technologies are opening up the possibilities of carrying out experiments that were inconceivable even 5 or 6 years ago, and it is certain that we are on the threshold of making major new breakthroughs in understanding the brain [24]. But then why the trepidation? The history of experimental neuroscience can be written as tales of very smart people who used extraordinary intuition to find meaning in the wealth of detail available to them. Different from today, many of the findings in the past were instantly recognizable once the experiment was done and were not hidden in model-dependent analyses. For example, when we saw, for the first time, serotonin-like staining in the crustacean stomatogastric nervous system [25], there were important controls and replications to be done, but the first image had in it the entirety of the essential finding. Undoubtedly there are still “reveals” of this sort that will come, as we continue to develop new microscopes and new reagents. But more often, the new findings will depend on data analyses that are highly quantitative and that employ statistics and algorithms that many of their users may not completely understand—hence, part of my trepidation. I look around at a new generation of biologists who are not just using voltage clamps that they didn’t build (and may not understand) but who are using data-analysis packages without necessarily understanding the assumptions and limitations in their analysis tools.

## The Importance of Intuition

It is very different to experience directly a new finding by looking at raw data, be they spike trains, gels, or electron micrographs, than to only see data after they are “crunched” with methods that are overtly or subtly model dependent. Under these conditions, intuition is even more valuable, but now the intuition plays the role of asking which kind of data treatment will give an answer that is true to the essence of the biological process studied. In a sense, one hopes that intuition will reveal which of those thousands of fascinating biological details are relevant to the problem at hand. I worry that we are on the threshold of generating a whole new class of “mistakes” that are outcomes of data analysis errors that arise when the answers are not immediately obvious from looking at the raw data and when inappropriate tools are inadvertently applied. The likelihood of errors in the experimental literature that arise from inappropriate or incorrectly applied statistical treatments has increased dramatically in recent years as we have started to consider the results of correlated measurements in gene expression, electrical activity, or features in behavior.

A deeper cause for my trepidation comes from the existence of parallel pathways and multiple solutions in large brains [13,26,27]. Detailed connectomes will assuredly show the extent to which neurons and brain regions are connected by multiple parallel pathways. While we can speculate that parallel pathways can provide additional robustness to circuits, or increase their dynamic range and flexibility, they make it far more difficult to predict the outcome of a specific pattern of activity or understand the results of a perturbation. As the number of parallel pathways and brain circuit loops increase, deducing how information flows through large and multiply interconnected circuits and why and under which contexts will be a challenge for many, many years to come.

In principle, theoretical and computational studies provide us the rigorous tools to extract understanding from seas of data by giving us perspective and helping us develop a new set of intuitions into what details matter for how the brain works [28]. However, many theorists entered neuroscience with the mind-set that big-picture solutions will be independent of the specifics of the implementation used in the brain to solve any given problem, and they therefore implicitly believe that most of the details about how the brain is built are irrelevant to how it computes. Sometimes they choose a specific favorite detail because it appears to potentially have a potential computational function and then build models that explore that function, but in unphysiological contexts. Of course, there are also experimentalists who fall in love with their favorite molecule and attribute all manner of function and dysfunction to that molecule without considering the larger context in which it acts.

Most theoretical studies use simplified model neurons [29] and circuit architectures, which, in principle, should lead us to see fundamental concepts more clearly. Nonetheless, the danger always remains that these simplifications and abstractions violate biological reality and result in studies that are mathematically true of the abstract formulism studied but not necessarily instructive of how brains work.

The other extreme, models that attempt to slavishly reproduce the brain’s structure, will be as difficult to understand as the brain itself, with the added disadvantage of being wrong, because all models, no matter how realistic, must involve choices of parameters, architectures, and simplifications. As models get more and more complex in an attempt to be more “realistic,” they inevitably become more wrong, as each added detail comes with associated approximations or unknowns.

Lest we wander for 40 years in a desert of our own creation, worshipping many false principles, it becomes imperative to create new and better methods for disciplined dimensionality reduction [30,31] that will allow us to build models that are true to the essential details in our biological data but provide big picture insight. Most theorists today would argue that they are already doing so; yet, a large fraction of experimental neuroscientists would be hard-pressed to provide specific examples of a model that influenced the design of their experiments. Certainly, there are significant findings that have come from theory about brain oscillations [32–35], energy efficiency [36,37], neural integrators [38,39], inhibitory/excitatory balance [40,41], noise, circuit degeneracy [13,42], and a host of other problems. There are increasing numbers of experiments that are designed with theoretical predictions in mind, but the gap between the theory and experimental communities remains entirely too large. Ironically, there are many examples of ideas that were first proposed theoretically that have become so second nature to the experimental community that their theoretical origins are often overlooked by today’s students, who now view them as “givens.”

Theoretical studies of brain function are most useful when they generate new intuitions [28] or show why a widely held belief is wrong. Crucially important are models that will help us develop intuitions into which biological details are significant for a given brain function and which details can, as a first approximation, be ignored. Some of those details may be critical for understanding how the same circuit of neurons behaves under different circumstances, different time scales, or in response to different perturbations. So, paradoxically, the future will depend on stronger quantitative understanding in our experimental community and better biological intuition in our theory community. Thus, enhancing conversation across our joint community provides the path to extracting general principles from the multitudes of precious details our brains exploit to generate humor, personality, curiosity, and all of the features we prize in our friends and loved ones.
